# Technical and Consumer Preferences Integrated for the Development of Cassava Varieties with High Nutritional Quality Adapted to Colombian Caribbean Coast

**DOI:** 10.3390/plants14213238

**Published:** 2025-10-22

**Authors:** Amparo Rosero, Hernán Ceballos, Rommel León, Jorge García, Alfonso Orozco, Gabriel Silva, Martha Montes, Remberto Martínez, Carina Cordero, Victor de la Ossa, Sonia Gallego-Castillo, Jorge Iván Lenis, Sandra Salazar, John Belalcazar, Wilson Barragán-Hernández

**Affiliations:** 1 Corporación Colombiana de Investigación Agropecuaria—AGROSAVIA, Centro de Investigación Obonuco, Pasto 520001, Colombia; 2 Alliance of Bioversity International and International Center for Tropical Agriculture—CIAT, The Americas HUB, Km 17 Recta Cali-Palmira, Palmira 763537, Colombia; 3 Corporación Colombiana de Investigación Agropecuaria—AGROSAVIA, Centro de Investigación Caribia, Zona Bananera 502041, Colombia; 4 Corporación Colombiana de Investigación Agropecuaria—AGROSAVIA, Centro de Investigación Turipaná, Cereté 250047, Colombia; 5 Corporación Colombiana de Investigación Agropecuaria—AGROSAVIA, Centro de Investigación Motilonia, Codazzi 202050, Colombia; 6 Corporación Colombiana de Investigación Agropecuaria—AGROSAVIA, Centro de Investigación Turipaná, Sede Carmen de Bolívar 250047, Colombia; 7 Corporación Colombiana de Investigación Agropecuaria—AGROSAVIA, Centro de Investigación El Nus, San Roque 053037, Colombia

**Keywords:** cassava, introgression, β-carotenes, yield, consumer’s preferences, food quality, selection index

## Abstract

Increasing the nutritional composition of food is a strategy to add added value to products in key agrochains, contributing to food security, and providing nutritional compounds available to improve selected nutritional deficiencies. An increased level of β-carotenes is an important contribution to reducing vitamin A deficiency. In Colombia, the Bioversity-CIAT alliance and Agrosavia evaluated eight cassava genotypes with the aim of identifying promising candidates for commercial release adapted to Caribbean region in Colombia. Experimental genotypes were established together with two checks, in six locations representing dry and humid Caribbean region. Agronomic evaluations, combined with culinary quality assessments and participatory evaluations of consumer preferences, enabled a comprehensive analysis of each genotype. The experimental genotypes exhibited different plant architecture, with some showing greater height and higher first branching than current varieties. However, excessive plant height in certain genotypes led to increased susceptibility to lodging, negatively affecting the quality of planting material. While only a few genotypes matched the check varieties in root yield (20 T/ha), several demonstrated significantly improved nutritional quality due to higher accumulation of total and β-carotenes (>8 µg/gr and >5 µg/gr, respectively) compared to current varieties (<2 µg/gr and 1.5 µg/gr, respectively). Cooking quality and consumer acceptance were key determinants in the final selection. Among the evaluated lines, genotype GM3426-5 stood out for its favorable agronomic performance, high provitamin A content, and excellent root and cooking quality. Nevertheless, further steps are required before its commercial release, as the product profile for cassava destined for human consumption must prioritize food quality and consumer preferences.

## 1. Introduction

Cassava (*Manihot esculenta* Crantz) is a major staple food crop cultivated across tropical and subtropical regions of the world [1]. It is grown on more than 33 million hectares globally, with about 75% of this area located in Africa [2]. Cassava roots provide a vital source of carbohydrates for over two billion people worldwide [3,4,5]. In addition to starch, cassava contains a range of bioactive nutrients, including minerals, essential fatty acids, antioxidants, and antidiabetic compounds [6].

Micronutrient malnutrition, often referred to as hidden hunger, affects more than half of the world’s population, particularly women and preschool-aged children. Globally, an estimated 163 million children are vitamin A deficient, with a prevalence of around 30% in developing countries [7]. In Colombia, micronutrient deficiencies also represent a major public health concern, with vitamin A deficiency especially widespread in certain regions. Young children are the most vulnerable: 28% of those aged 12–23 months and about one-quarter of children aged 1–4 years are vitamin A deficient, along with many women in reproductive age living in rural areas [8,9,10].

The core of malnutrition is a deficient diet and a key and sustainable strategy to overcome malnutrition is to improve the quality of diets. The development of new food products with high nutritional value (biofortification) can be achieved through conven-tional breeding approaches [11,12,13]. The global biofortification index (BPI), which is based on three sub-indices related to pro-duction, consumption and micronutritional deficiency, established that crops biofortified with vitamin A should be introduced on the Atlantic Coast and Amazonas regions of Colombia [14,15]. Although, there is a wide varietal diversity in cassava crop, in Colombia, most consumed varieties are white or cream flesh color, which composition present high percentage of dry matter and more than 80% of it are carbohydrates [16].

The root cause of malnutrition is a poor-quality diet, and a key sustainable strategy to address it is to improve the quality of diets. One effective approach is the development of crop varieties with enhanced nutritional value (biofortification), which can be achieved using conventional breeding methods [11,12,13]. The Global Biofortification Index —which integrates three sub-indices related to production, consumption, and micronutrient deficiency—identified the Atlantic Coast and Ama-zonas regions of Colombia as priority areas for the introduction of vitamin A–biofortified crops [14,15]. Despite the wide varietal diversity of cassava in Colombia, the most commonly consumed varieties have white or cream-colored flesh, characterized by a high dry matter content, of which more than 80% consists of carbohydrates [16]. These varieties, however, provide only minimal amounts of micronutrients.

In the case of vitamin A, provitamin A (pVA) carotenoids are the primary precursors supplied by food plants. Carotenoids are natural lipophilic isoprenoids responsible for the yellow to orange pigmentation in plants. They play a key role in photosynthesis and act as precursors for phytohormones and signaling molecules essential for plant homeostasis [17]. The main carotenoids with provitamin A activity are β-carotene, α-carotene, and β-cryptoxanthin, with β-carotene being one of the most abundant carotenoids in nature [18]. For decades, cyanogenic glucosides have been the primary—if not the only— nutritional concern associated with cassava roots [7,19]. Early reports of increased protein content [20] later proved misleading. Efforts to enhance other nutritional traits, such as higher zinc or iron levels, have been attempted through genetic transformation [21], but no varieties with these improvements have been released. By contrast, the successful enhancement of pVA carotenoid levels has been demonstrated, and varieties with this trait are now being gradually released worldwide. These efforts are expected to contribute to reducing health problems associated with vita-min A deficiency [22,23,24].

However, some challenges for the successful deployment of biofortified cassava vari-eties with high pVA have been acknowledged [25]. Although substantial progress has been made in increasing carotenoid levels in cassava roots, the agronomic performance and consumer acceptance of these varieties still need to be fully evaluated. The objective of this study, therefore, was to evaluate the agronomic, nutritional and cooking quality characteristics of cassava genotypes selected for their high pVA carotenoid content as well as consumer preferences.

## 2. Materials and Methods

### 2.1. Environmental Conditions

The Colombian regulations for the development of new varieties includes the Agro-nomic Evaluation Tests (AETs). Cassava genotypes were established during 2018–2019 in AETs grown in six localities in the Colombian Caribbean coast (Table 1). Detailed descrip-tion of the evaluation process from the seedling stage through the release of varieties is available [26].

### 2.2. Plant Material

Eight genotypes were selected after ten years of evaluations in different locations across the Dry and the Humid Caribbean coast of Colombia (CCC) in the Córdoba, Sucre, Atlántico and Magdalena Departments. Data originated in single row trials (unreplicated) and replicated preliminary, advanced, and uniform or regional trials [27].

The eight experimental genotypes (GM3518-42, GM3518-66, GM5194-13, GM3426-5, GM5146-19, GM5177-3, SM3767-84 and SM3536-44) possess high nutritional quality due to its high carotenoids content. They were compared with two commercial checks (Costeña and Venezolana). Venezolana is a traditional landrace variety widely cultivated on the CCC. It is identified as MCOL2215 in CIAT’s germplasm collection. This collection includes more than 5000 cassava accessions. ICA-Costeña is an improved variety derived from the cross between two landraces (MMEX 11 and MCOL 65) from the germplasm collection [26].

**Figure 1:**
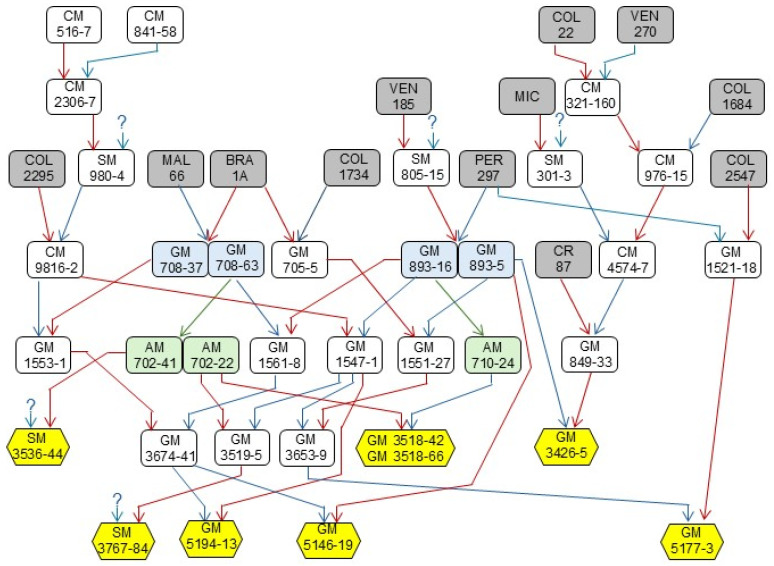
Pedigree of the eight experimental clones evaluated in this study (yellow hexagons). Red and blue arrows indicate mother and father relationships. Green arrows indicate self-pollinations. Grey cells highlight landraces from the germplasm collection. Blue cells identify two cases of siblings from the same family. Green cells highlight S_1_ families (AM code). GM and CM codes identify full-sib families. SM codes stand for half-sib families obtained from open pollinations (unknown male progenitor represented by ? symbol).

The experimental genotypes were obtained at CIAT cassava breeding program, fol-lowing the process to generate new improved varieties [26]. A rapid-cycling recurrent selection approach was implemented to increase the levels of carotenoids content which was the primary breeding objective [25,27]. Six experimental clones (GM3518-42, GM3518-66, GM5194-13, GM3426-5, GM5146-19 and GM5177-3) were obtained from controlled pollinations with known male and female progenitors (Figure 1). GM3518-42 and GM3518-66 are siblings from the same full-sib family. The two remaining experimental genotypes (SM3767-84 and SM3536-44) were obtained from open pollinations and thus, only the female progenitor is known. The known progenitors of the eight experimental clones were mainly selected for their high pVA carotenoid content introgressed from landraces in the germplasm collection. There was no agronomically outstanding germplasm in the ancestry in any of the eight experimental clones.

### 2.3. Experimental Design and Crop Management

The experimental design was under randomized complete block design (RCBD) with three replications. Each plot consisted of five rows, each measuring five meters in length, with a planting density of 10,000 plants per hectare (spaced at 1 m between rows and 1 m within rows), nine central plants were used as a useful plot, discarding from the evaluations the plants with border effect. Soil preparation and planting were carried out according to the edaphological and agroclimatic conditions of each zone, weed management included the application of pre-emergent herbicides, graminicides, and manually. The fertilization was done according to the results of the soil analysis and considering the management that the farmer conventionally offers to the crop.

### 2.4. Evaluation of Agronomic Traits

Total and commercial fresh root yield (FRY) was calculated in t/ha from parameters taken on a plot basis (commercial and total root weight), dry matter content (DMC) was calculated according to the standard gravimetric method.

Several variables related to plant establishment and architecture were measured. Germination percentage refers to the number of stakes that sprouted one month after planting. Vigor was evaluated between two and four months after planting, using a scale from 1 to 5, where 1 represents very low vigor and 5 represents very vigorous growth.

Plant height and height to the first branching were measured in centimeters, from the soil surface to the topmost part and to the first branching point, respectively. The number of branch levels corresponds to the number of distinct branching levels on the plant.

The number of stakes per plant was estimated by counting potential planting stakes (cuttings) of good quality, defined as 20 cm in length with at least 5 nodes. Plant type was assessed on a scale from 1 to 5, where 1 corresponds to the ideal plant type in terms of architecture, vigor, and overall plant health, and 5 represents plants clearly performing below average. Lastly, lodging was evaluated using a scale from 1 to 3, where 1 indicates no or very little lodging, 2 indicates intermediate lodging, and 3 indicates severe lodging.

### 2.5. Evaluation of Root Quality Traits

Cooking time, texture and friability were measured to assess cooking quality of roots after boiling [28,29]. The following procedure was used: (i) Two or three representative and healthy plants were selected and pooled out of the ground with special efforts to prevent root damages; (ii) individual roots from the crown of roots of each plant were carefully separated and classified as commercial (large or medium-sized roots, with a diameter > 5 cm), non-commercial (small roots, with a diameter < 5 cm) and fibrous (very small and thin roots, with a diameter < 2 cm); (iii) roots were washed with tap water and allowed to dry in open air conditions. Commercial, non-commercial and fibrous pools of roots were weighed; (iv) commercial roots were then peeled and cut into 1–2 cylinders (6 cm long) from the central part; (v) break each cylinder into two pieces, discard the fibrous central core and weigh the remaining pieces in a previously identified mesh bag; (vi) pieces were then immersed into water already boiling in a pot; (vii) after 10 min, a fork was inserted into the pieces to assess its consistency (ideally, the fork must penetrate gently). This task was carried out every 5 min until the pieces had the right texture (firm and soft); (viii) boiled pieces were allowed to cool for a while and weighed; (ix) culinary quality of the cooked pieces was evaluated by a panel for texture (hard/glassy, soft, firm), flavor (bitter, neutral, sweet) and fiber content (acceptable, non-acceptable).

A hedonic scale was used to sensorially assess the cooking quality of roots from different genotypes. The scale had five categories ranging from “extremely dislike” to “extremely like”. Cassava consumers (n = 90) were recruited to evaluate roots from all experimental clones and the two checks. All genotypes were renamed with randomized codes. Participants were asked to evaluate the color, flavor, and texture for each sample and also received a glass with ambient temperature water to cleanse their mouth between sample testing. Finally, a whole representative root from each genotype was exhibited to be evaluated and scored according to the root shape and other morphological traits that end users take into consideration.

**Figure 2:**
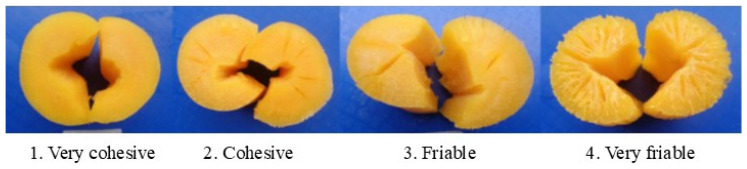
Contrasting appearances of root cylinders after boiling.

After cooking, the appearance of the cylinder was also evaluated according to the characteristic “friability” (Figure 2), which is related to the tendency of cassava to crack after the cooking process [30].

Total carotenoids (TCCs) and β-carotene (TBC) were predicted on uncooked fresh roots through a well-established protocol using near infrared spectroscopy [31]. Cyanogenic potential (HCN) was determined according to the method described by Essers et al. (1993) [32].

### 2.6. Statistical Analysis

The collected data was analyzed according to a multilocal experiment, as described by [33,34]. An analysis of variation was performed as mixed model that considered the fixed effects of the genotype, the region and the interaction between these factors. Random effects were localities within each region and blocks within each locality. The Equation (1) describes the statistical model

(1) $$y_{ijkn}=\mu +G_{i}+R_{j}+L_{n}+B_{k(jn)}+{GR}_{ij}+e_{ijkn}$$

$y_{ijkn}$ = Measured random variable
μ = General average
$G_{i}$ =Effect i-th genotype

$R_{j}=Effect\;\mathrm{j-th}\;locality$

$L_{n}=Effect\;\mathrm{n-th}\;locality\;idd\;N(0,\sigma_{l}^{2})$

$B_{k(jn)}=Effect\;\mathrm{k-th}\;block\;idd\;N(0,\sigma_{b}^{2})$

${GR}_{ij}=Effect\;genotype\;x\;locality\;interaction$

$e_{ijkn}=Experimental\;error\;idd\;N(0,\sigma^{2})$

For the analysis of variables associated with content, percentages or variable pre-sented a distribution of residuals different from normal, the data was analyzed with a generalized ANOVA, using a distribution of residuals appropriate to the nature of the variables, such as Poisson, Beta, Gamma, among others. Obtained *p*-values for all factors and variables were reported. For variables with ordinal qualitative nature for both agro-nomic and sensorial evaluation, data analysis was performed as described by Agresti (2012) [35]. This model calculates the cumulative probability, (P(Yi ≤ j), which represents the likelihood that an observation (i) belongs to category (j) or a category with a smaller value.

Analysis was carried out using the R-Project software, employing appropriate pack-ages for each analysis such as lme4 [36] and ordial [37], in addition to support packages for analysis and visualization. In all analyses, an alpha of 0.05 was considered and in case of rejection of the null hypothesis, a Tukey test was used with correction for multiple comparisons.

## 3. Results

### 3.1. Plant Architecture and Morphological Features

Analysis of variance revealed significant differences among genotypes for all plant architecture traits (Table 2). Differences between the Humid and Dry Sub-regions were also significant, except for height of first branch and number of branching levels. Genotype-by-environment interaction was significant for all traits except sprouting (Table 2). Chi-square logistic regression for qualitative variables showed significant differences among genotypes for vigor, plant type, and lodging, with plant type being the only variable that exhibited a genotype-by-Sub-region interaction.

**Figure 3:**
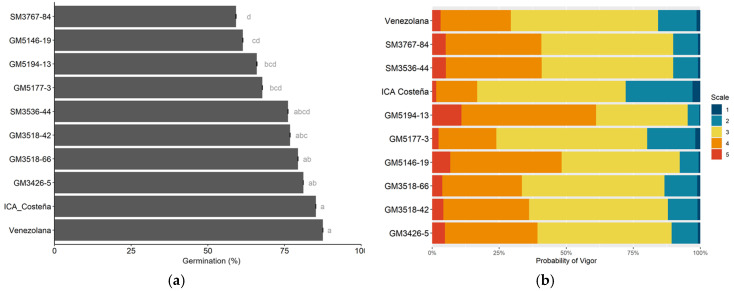
(**a**). Sprouting percentage. (**b**). plant vigor (right). Different letters indicate significant differences determined by Tukey’s test (*p* < 0.05).

The highest sprouting after planting was observed in the genotypes used as controls, and four experimental clones had significantly lower sprouting percentage (Figure 3). GM3426-5 and GM3518-66 presented the highest average within the eight experimental genotypes. Regarding plant vigor, the chi-square test showed significance in the relationship between vigor and genotypes. A cumulative probability > 75% rated vigor on scales ≥ 3 in all evaluated genotypes. These scores identify desirable vigor phenotypes and are comparable with the most vigorous control (Venezolana) (Figure 3b). No associations were found between vigor and locations.

**Figure 4:**
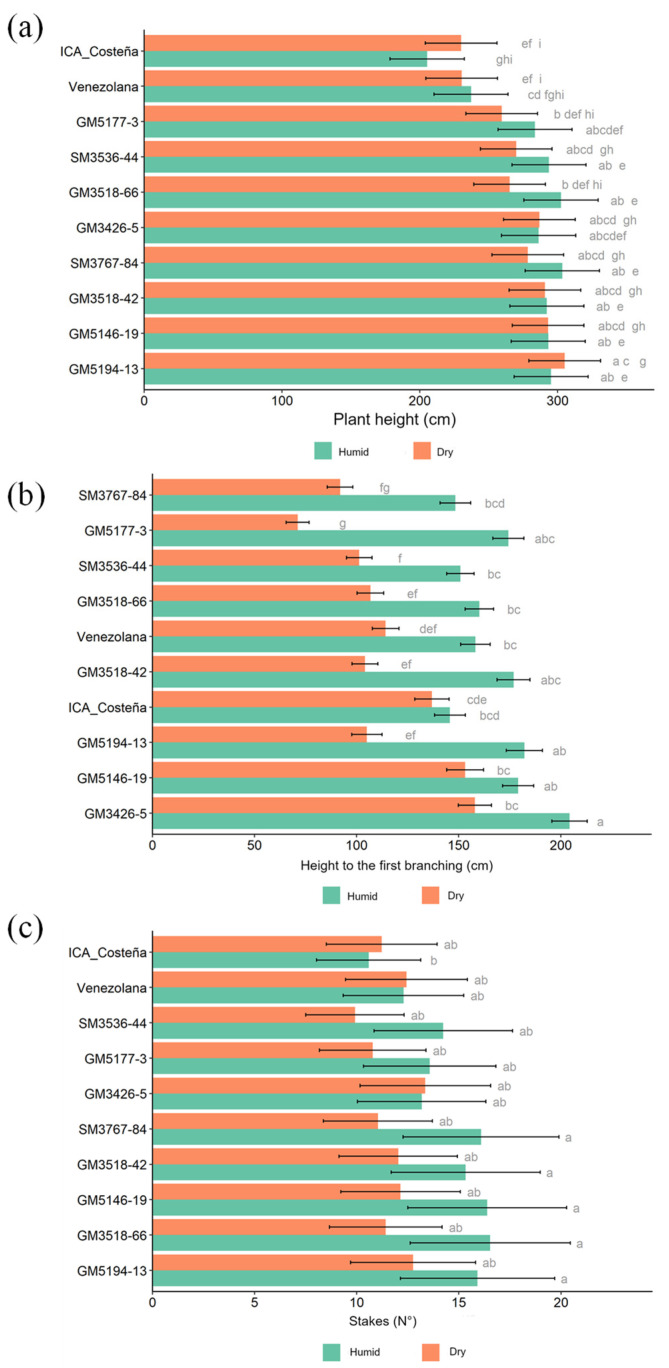
Agronomic performance for key plant architecture traits. (**a**). Plant height (cm); (**b**). Height of first branching (cm); and (**c**). Number of stem cuttings per plant. Different letters indicate significant differences determined by Tukey’s test (*p* < 0.05).

The experimental genotypes exhibited greater plant height than the controls in both the Dry and Humid Caribbean Sub-regions (Figure 4a). The height of the first branching event was consistently and substantially higher in the Humid than in the Dry Sub-region (Figure 4b). In the Humid Sub-region, differences among genotypes were generally small and reached statistical significance only in a few cases. Importantly, all genotypes branched at more than 150 cm above ground, which is desirable. Under Dry conditions, however, the height of first branching varied widely among genotypes, with GM5177-3 showing an unacceptably low value.

Under Humid conditions, most genotypes exhibited about two branching events, with little variation among them. In contrast, under Dry conditions, genotypes GM5194-13, GM5146-19, GM5177-3, SM3787-84, and SM3536-44 displayed more than three branching levels. Interestingly, the check genotypes produced more stem cuttings per plant under Dry conditions (Figure 4c), whereas the experimental clones tended to produce more under Humid conditions. Overall, however, the number of cuttings was similar among genotypes and considered acceptable from an agronomic perspective.

**Figure 5:**
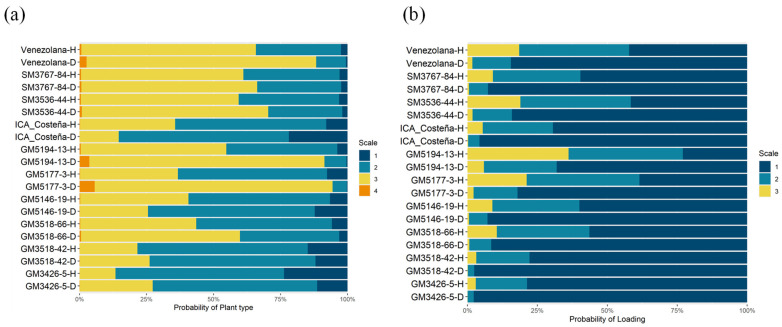
Agronomic performance for key plant architecture traits. (**a**). Plant type score; (**b**). Lodging. D: dry Caribe, H: humid Caribe.

The plant type score provides an overall assessment of plant architecture, vigor, health, and leaf retention at harvest. Lower scores indicate a better plant type. Genotypes GM5177-3, SM3767-84, SM3536-44, and GM5194-13, along with the check Venezolana, had a probability greater than 50% of falling into the undesirable categories 3 and 4 (Figure 5a). In contrast, genotypes GM3426-5, GM3518-42, and GM5146-19 showed a higher probability of being classified in categories 1 and 2, which are desirable and comparable to the remaining check, ICA-Costeña.

In the Humid Sub-region, lodging was observed as early as seven months after planting. Although its frequency was not high, lodging can affect the quality of planting material for the next crop cycle and complicate harvesting (Figure 5b). Genotypes GM3426-5, GM3518-42, GM3518-66, GM5146-19, and SM3767-84 showed a lower probability of lodging, comparable to the ICA-Costeña control and lower than that observed in the Venezolana landrace.

### 3.2. Root Yield and Dry Matter Content

Analysis of variables related to root yield and dry matter content (DMC) revealed significant differences (*p* = 0.01) among genotypes for number of commercial-size roots per plant (NCR), total fresh root yield, and DMC (Table 3). No significant differences were detected between regions for any variable. However, the genotype × region interaction was significant (*p* = 0.01) for NCR and for the weight of commercial roots per plant.

**Figure 6:**
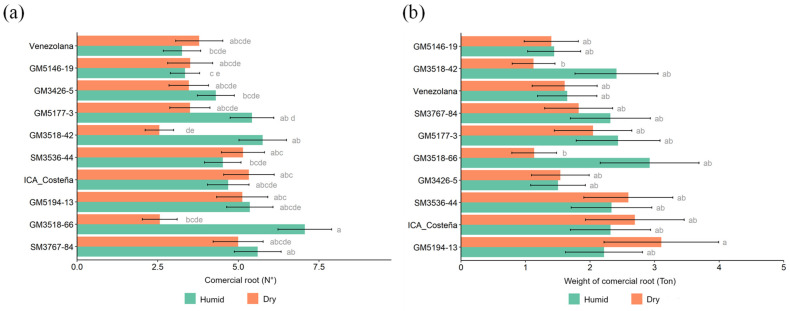
(**a**). Number of commercial-size root yield per plant; (**b**). Weight of commercial-size root yield (kg/plant). Different letters indicate significant differences determined by Tukey’s test (*p* < 0.05).

For the production of commercial roots per plant, significantly higher values were generally observed under Humid conditions. Genotypes SM3536-44, ICA-Costeña, GM5194-13, and SM3767-84 produced more than five roots per plant in both environments (Figure 6a). Wide variation was observed in the weight of commercial-size roots, with GM3518-42 and GM3518-66 showing significantly lower values in the Dry Sub-region. The remaining genotypes performed statistically similar to the controls (Figure 6b).

**Figure 7:**
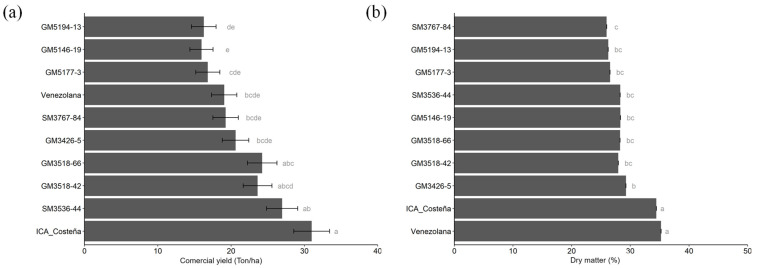
(**a**). Commercial-size root yield (t/ha). (**b**). Root dry matter content (%) by the gravimetric method. Different letters indicate significant differences determined by Tukey’s test (*p* < 0.05).

Several genotypes produced a higher commercial-size root yield than the check Venezolana; however, none surpassed the ICA-Costeña control (Figure 7a). The commercial yield of SM3536-44 was significantly higher than that of Venezolana. Experimental genotypes generally showed significantly lower DNC than the two checks, although GM3426-5 had an average close to that of the controls.

### 3.3. Root Quality Traits

**Figure 8:**
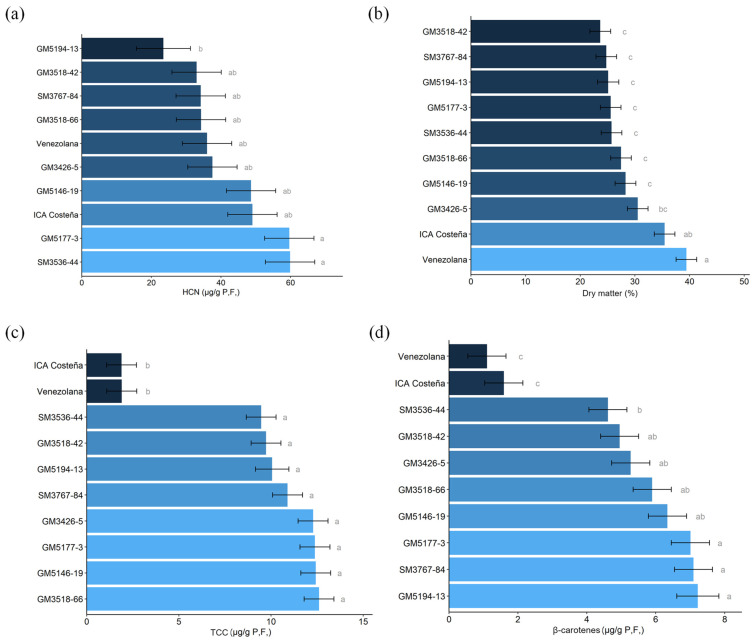
Root quality parameters. (**a**). Cyanogenic potential (HCN); (**b**). Dry matter content measured by the oven-drying method; (**c**). Total carotenoids content by NIRs; (**d**). Total β-carotene by NIRs. Different letters indicate significant differences determined by Tukey’s test (*p* < 0.05).

Significant differences were detected among genotypes for all root quality traits, but not between Sub-regions or for the Sub-region × genotype interaction (Table 4). All genotypes exhibited low cyanogenic potential (HCN) on a fresh weight basis, well below the maximum acceptable threshold for taste and health (Figure 8a). However, SM3536-44 and GM5177-3 averaged around 60 µg/g, a level that may affect cassava flavor. The oven drying method estimates confirmed that experimental genotypes generally had significantly lower DMC than the two controls, except for GM3426-5, which reached nearly 30% (Figure 8b). Experimental genotypes significantly exceeded the controls in total carotenoids (TCC) and total β-carotene (TβC), while the checks did not surpass 2 µg/g TCC and remained below 2 µg/g TβC (Figure 8c,d). No significant environmental effects were observed between the Dry and Humid Sub-regions, suggesting relatively high heritability for these traits.

### 3.4. Cooking Characteristics and Consumers Acceptability

**Figure 9:**
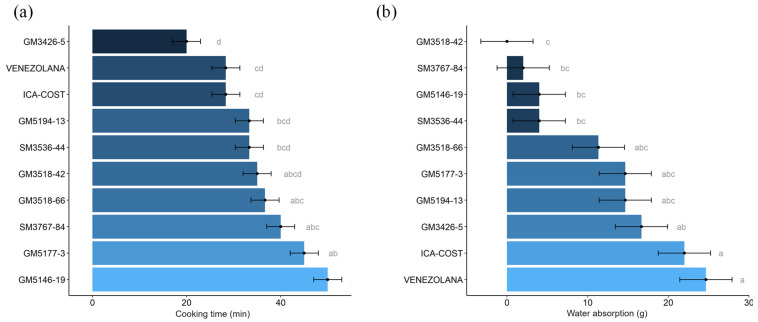
Cooking time (**a**) and water absorption (**b**) of roots from the evaluated genotypes. Different letters indicate significant differences determined by Tukey’s test (*p* < 0.05).

Optimal cooking time was measured from the immersion of root half-cylinder sec-tions in boiling water until a consistency between firm and soft was reached. Genotypes varied in their response, with some showing a favorable profile for cooking, ranging from 20 min (GM3426-5) to around 30 min (Venezolana, ICA-Costeña) (Figure 9a). Similarly, the increase in weight of boiled cylinders due to water absorption was highest in Venezo-lana, ICA-Costeña, GM3426-5, GM5194-13, and GM5177-3. The previously reported nega-tive correlation between cooking time and water absorption [3] was generally confirmed (Figure 9a,b), except for GM3536-44, which exhibited unexpectedly low water absorption. Experimental genotypes GM3426-5, GM5194-13, and GM5177-3 showed weight gain comparable to the checks, demonstrating good water absorption during cooking.

**Figure 10:**
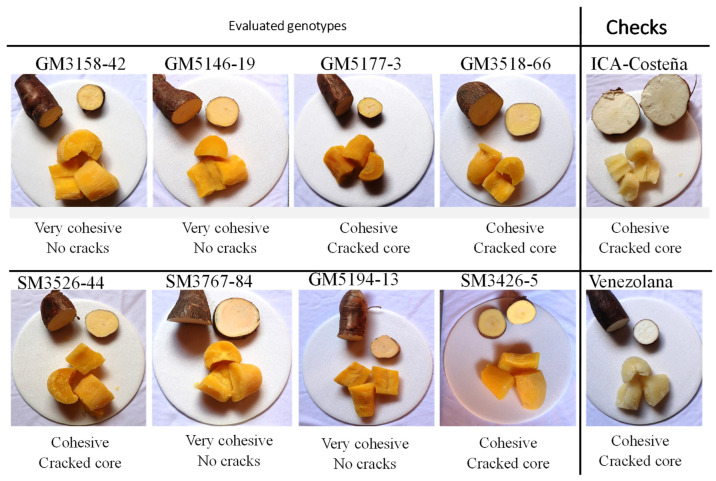
Appearance of root half-cylinders of the ten genotypes after boiling. The two samples on the right are the commercial checks.

In terms of friability, levels 2 (cohesive) and 3 (friable), which are also associated with adequate texture, are the most desirable by the consumer. Roots from four experimental genotypes were very cohesive after boiling (Figure 10). Genotypes GM3426-5, GM3518-66, GM5177-3 and SM3536-44, presented a consumer preferred cohesive texture which was comparable to the checks.

Finally, to assess the acceptance of the new cassava genotypes by consumers, sensory tests were conducted in Codazzi, Sevilla, and Cereté using a hedonic scale. The genotypes were cooked following the previously standardized procedure, and randomized codes were assigned to each genotype to avoid bias. Sensory responses were independent of the tasters’ gender (Table 5). However, a significant relationship was observed between location and product perception, suggesting cultural preferences in certain areas for yellow cassava and potential environmental effects on root quality (Table 5). Additionally, a significant relationship was found between genotypes and consumer-perceived characteristics, including flavor, texture, and external root shape (Table 5).

Regarding the localities, tasters in Cereté generally had a more favorable perception of yellow genotypes in terms of color, flavor, texture, and external shape, with most responses scoring 4 or 5 (“like a little” and “like it,” respectively) (Table 6). In contrast, at Codazzi and Sevilla, only the perception of external root shape frequently scored 3 or higher. For flavor and texture, the highest frequency of responses was concentrated at score 2, followed by 4, indicating a highly contrasting opinion of yellow cassava genotypes among the population. Regarding color, tasters in Codazzi showed low acceptance of the yellow color of the evaluated genotypes, which likely influenced their overall perception.

**Figure 11:**
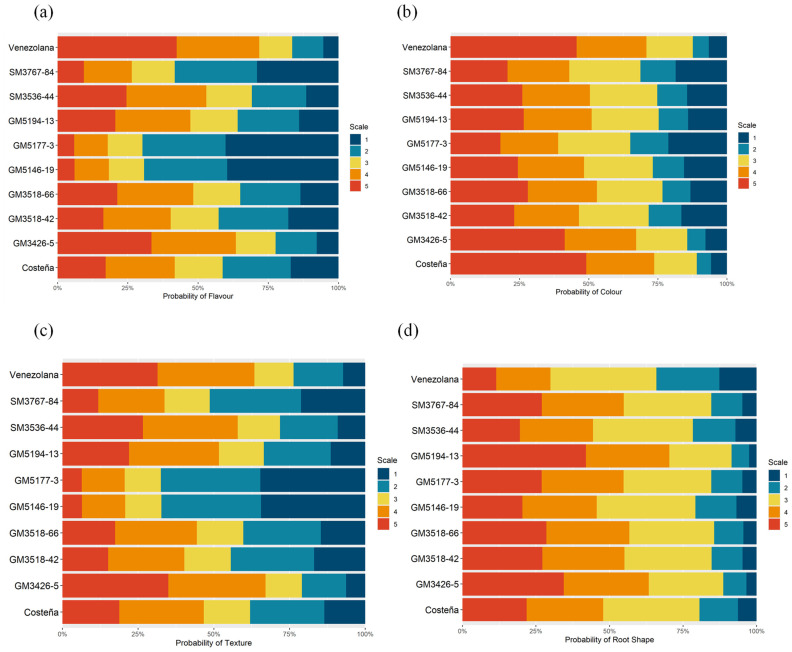
Probability of qualifications according to the hedonic test. (**a**). flavour; (**b**). colour; (**c**). texture; (**d**). root shape.

Consumer perception favored genotype GM3426-5 for flavor, with a probability greater than 75% of being classified in categories 3–5, comparable to the Venezuelan control, which is widely accepted for its culinary and flavor qualities after cooking (Figure 11a). For color after cooking, GM3426-5 also showed a high probability of being rated in categories 3–5, similar to the controls (Figure 11b). Likewise, for texture, GM3426-5 had a high probability of receiving scores of 3–5, comparable to Venezolana (Figure 11c). Overall, sensory tests with untrained tasters indicated good acceptance of GM3426-5, which consistently achieved a higher probability of scores ranging from 3 to 5 across all evaluated traits, including root shape (Figure 11d).

Finally, the sensory quality profile of genotype GM3426-5 was compared with all evaluated genotypes. Logistic regression analysis revealed significant differences between GM3426-5 and the lowest-rated genotypes (Table 7), confirming its superior culinary quality.

## 4. Discussion

Introgression of simple inheritance traits is a relatively recent strategy in cassava breeding. The specific traits that have been incorporated into released varieties include re-sistance to whiteflies from the landrace ECU72 [38], dominant resistance to cassava mo-saic disease [39], recessive amylose-free starch, and the high carotenoid content described in this study [40]. These experiences offer new insights into the unforeseen challenges of trait introgression using both conventional and genomic breeding approaches [25].

### 4.1. Plant Architecture and Morphological Features in Evaluated Cassava Genotypes

All plant architecture and morphological traits were significantly influenced by gen-otypic variation and, in most cases, also by significant genotype × environment interac-tions (Table 2). These findings are consistent with previous reports [41,42].

The overall evaluation of plant type implies that excessive plant height is undesirable, as it is associated with increased susceptibility to lodging, which in turn can promote early sprouting in pre-harvest plants [42]. This effect was more pronounced in locations within the Humid Caribbean Sub-region (Figure 5b). The experimental materials evaluated in this study do not include improved germplasm with outstanding agronomic performance (Figure 1), so it is not surprising that some clones exhibited deficiencies. All experimental clones displayed greater, though not excessive, plant height compared with the checks (Figure 4a). However, several clones (GM3518-42, GM5146-19, and GM3426-5) showed acceptable height of first branching (Figure 4b), comparable to or exceeding that of the checks. This outcome aligns with expectations, as breeders apply strong selection pressure against early branching, and the trait has high heritability.

In the early stages of selection, it became evident that germplasm with increased root carotenoids often showed poor sprouting, with many segregating progenies failing to sprout entirely. Although the experimental clones evaluated in this study were selected for rapid and high-percentage sprouting, their sprouting rates were still lower than those of the checks (Figure 3a). In particular, SM3767-84, GM5146-19, GM5194-13, and GM5177-3 exhibited significantly lower sprouting compared with the commercial checks.

A generalized lack of sprouting capacity is uncommon in cassava, as most breeding populations sprout adequately when planting material is properly stored. However, biofortified germplasm displayed an unexpected weakness in this regard. During the early stages of selection, it was hypothesized that poor sprouting could result from a lack of adaptation related to the ancestry of the progenitors involved (Figure 1). If this were the case, there might be potential to break the genetic linkage(s) between high carotenoid content and deficient sprouting. Nevertheless, increased carotenoid concentration in cassava has already been associated with unforseen effects on other aspects of plant metabolism, including stress tolerance and variation in levels of phytohormones (e.g., abscisic acid), fatty acids, triacylglycerols, and soluble sugars [43,44,45]. Reduced sprouting in biofortified cassava may, therefore, represent another pleiotropic effect of elevated carotenoid levels.

### 4.2. Agronomic Performance of Evaluated Genotypes

The number and weight of commercial roots showed significant genotype × environ-ment interactions, with some genotypes performing better under subhumid conditions, suggesting specific adaptation to these environments. Under Humid conditions, genotypes GM3518-42, SM3767-84, GM5177-3, and GM3518-66 exhibited higher numbers and weights of commercial roots compared with their averages under subhumid conditions. Similar contrasting results have been reported previously [46,47].

Average root yield of commercial roots across environments ranged from 16 to 27 t/ha in the eight experimental genotypes, whereas the respective averages for Venezolana and Costeña were, 19 and 31 t/ha, respectively (Figure 7a), and it was not affected by en-vironment neither their interaction with genotype (Table 3). The results in this study showed differences in commercial root yield mainly attributed to genotypes, consistently with moderate heritability (78%) reported in pro-vitamin A populations [47].

Multilocation evaluation is essential for the proper selection of promising genotypes, enabling the identification of wide and narrow adaptability [42]. While some genotypes showed reduced yields under stress, others performed well across different stressful condi-tions and phenological stages, indicating potential adaptation to dry environments [48].

DMC was primarily determined by genetic factors, with negligible influence from the environment or genotype × environment interaction (Table 3). This study therefore con-firms that DMC is a relatively stable trait, consistent with previous reports in pVA popula-tions, which showed high heritability (87%) [47]. High and stable DMC remains a key ob-jective of cassava breeding programs. In field evaluations, GM3426-5 was the top-performing experimental genotype, with a DMC of approximately 30%.

Figure 7b provides evidence of another pleiotropic effect of high carotenoid content in cassava roots: a clear tendency toward reduced DMC. This trend was also observed when DMC was measured by oven drying (Figure 8b). Similar observations can be derived from different publications [25,43]. Glucose-6-phosphate (G6P) and/or glucose-1-phosphate (G1P) transported into the amyloplast can be directed either toward starch synthesis or toward carotenoid and fatty acid synthesis [45]. This provides a plausible explanation for the negative correlation between carotenoid content and DMC observed in this study. Recent evidence supports the hypothesis that pleiotropy, rather than genetic linkage, underlies the negative genetic correlation between carotenoid content and DMC [49]. This find-ing reduces the likelihood of overcoming the tendency of biofortified cassava to exhibit lower-than-desirable DMC. The lower DMC in high pVA cassava roots may also partially explain their reported delayed onset of post-harvest physiological deterioration [43,50].

### 4.3. Root Quality Characteristics and Consumers Acceptability

Some genotypes exhibited yield values comparable to the top-performing check, ICA-Costeña. However, given the importance of consumer acceptance, selection should not focus solely on yield and agronomic traits; cooking quality must also be considered [51]. For boiled cassava, the market demands varieties with good culinary quality, low cyanogenic potential, and high dry matter content [16,28].

Non-bitter cassava roots typically contain less than 100 μg/g cyanogenic glucosides on a fresh weight basis [52,53], and concentrations below 50 μg/g are considered safe for consumption [19]. Several experimental genotypes exhibited HCN levels below the aver-age of ICA-Costeña, indicating they are non-bitter and, importantly, safe to eat (Figure 8a). Among these was the promising clone GM3426-5.

In this study, cooking quality parameters tended to be better in genotypes with higher DMC. The relationship between cooking time and water absorption confirmed previous reports [3,52]. In addition to its acceptable DMC, GM3426-5 exhibited excellent cooking time, the associated water absorption (Figure 9), and a cohesive texture (Figure 10). Proba-bility analysis of sensory data showed that GM3426-5 was preferred by panelists for color, flavor, texture, and root shape among the experimental clones (Figure 10 and Figure 11). In con-trast, genotypes GM3518-42, GM5146-19, GM5194-13, and SM3767-84 displayed very co-hesive texture (Figure 10), poor scores for flavor and texture (Figure 11), and excessive cooking time (Figure 9).

Variety adoption depends not only on agronomic performance but, more importantly, on quality traits that determine consumer acceptance [51]. The sensory evaluation of cas-sava genotypes by untrained panelists contributed to the selection of promising clones. A hedonic scale was used to assess color, flavor, texture, and root shape. Chi-square analysis indicated that panelist gender did not influence sensory scores. However, consumer per-ception varied across the three locations (Table 6), suggesting that social and cultural fac-tors may affect the potential acceptance of yellow cassava varieties.

This study was undertaken in direct response to the reported high prevalence of vit-amin A deficiency in some regions of Colombia [8,10]. The experimental genotypes demonstrated significant improvements in provitamin A carotene accumulation com-pared with the checks. However, thorough screening of the biofortified genotypes revealed unexpected drawbacks, including reduced sprouting of stem cuttings after planting and lower DMC. Incorporating consumer preferences and quality parameters related to cook-ing guided the appropriate selection of promising clones. Biofortified cassava varieties have already been released in Brazil and Africa and are expected to contribute to reducing health problems associated with vitamin A deficiency [22,23,24].

## 5. Conclusions

The integration of agronomic evaluation with participatory assessments of product profiles, both as a crop and as food, provides a comprehensive framework for genotype selection in cassava breeding. This study demonstrated successful introgression of high carotene content; however, observed differences in plant architecture and sprouting compared to commercial cultivars highlight the need for continued improvement of these traits. Among the evaluated genotypes, the experimental clone GM3426-5 emerged as particularly promising, combining high provitamin A carotenoid content with strong agronomic performance, good yield, and desirable root and cooking qualities. On Colombia’s Caribbean coast, where white-fleshed cassava is traditionally preferred, targeted marketing strategies will be essential to support consumer acceptance of biofortified varieties. Future cassava breeding efforts should continue to address the challenges associated with trait introgression to ensure the development of varieties that meet both agronomic and consumer demands.

## Figures and Tables

**Table 1:** Main characteristics of localities used in this study.

N°	Landscape	Municipality	Additional Information
Humid Sub-region			
1	Flat savanna	Cereté	Córdoba Departament. Experimental field AGROSAVIA—C.I. Turipana (km 13 Vía Montería-Cereté), N 8°50′27.47″, W 75°48′27.56″, 12 m above sea level (m.a.s.l.)
2	Hilly/Piedmont	Carmen de Bolívar	Bolivar Departament. Experimental field AGROSAVIA—Sede Carmen de Bolívar, N 9°42′50.8″, W 75°06′26.9″, 197 m.a.s.l.
3	Flat savanna	La Unión	Sucre Departament. El Litoral Farm, Vereda El Paisaje. 8°48′13.2″ N 75°15′55.9″ W, 49 m.a.s.l.
Dry Sub-region			
4	Piedmont	Agustín Codazzi	Cesar Departament. Experimental field AGROSAVIA—CI Motilonia (km 5 vía a Becerril), 10°00′01.2″ N, 73°15′22.4″ W, 131 m.a.s.l.
5	Flat savanna	Sevilla (Banana production region)	Magdalena Departament. Experimental field AGROSAVIA—C.I. Caribia. Ubicado en Sevilla, Zona Bananera (Magdalena), N 10°47′32.081″; W 74°10′15.493″, 18 m.a.s.l.
6	Flat savanna	Caracolí	Atlántico Departament. Las Torres Farm. 10°49′53,5908″ N 74°52′14,5956″ W, 15 m.a.s.l.

**Table 2:** *p*-values obtained by Anova and Chi square analysis of plant architecture variables.

Factor	Genotype	Sub-Region	Genotypex Subregion
*p* values Anova analysis			
Sprouting	<0.0001	0.012	0.262
Plant height	<0.0001	0.826	0.018
Height of 1st Branch	<0.0001	0.000	<0.0001
Number of branching levels	<0.0001	0.006	<0.0001
Number of stem cuttings/plant	<0.0001	0.969	0.005
Logist regression values Chi square analysis			
Vigor	24.58 **	0.964	14.73
Plant type	117.40 ***	0.182	50.53 ***
Loding	75.53 ***	4.880	-

** indicated *p* value less than 0.001, *** indicated *p* value less than 0.0001.

**Table 3:** Analysis of variance (*p*-values) of relevant root yield components.

Source of Variation	Commercial Size Roots		Yield (t/ha)		Dry Matter
	(Number/Plant)	(kg/Plant)	Total	Commercial	Content (%)
Genotype (G)	0.0001	0.0361	<0.0001	0.07402	<0.0001
Region (R)	0.3264	0.9535	0.3261	0.42872	0.5987
GxR	0.0073	0.0048	0.1255	0.06396	0.1709

**Table 4:** Analysis of variance (*p* values) of root quality traits.

Source of Variation	Oven DMC	HCN	TCC-NIRs	TβC-NIRs
	(%)	(μg/g fw)	(μg/g fw)	(μg/g fw)
Genotype	<0.0001	0.0017	<0.0001	<0.0001
Sub-region	0.243	0.920	0.099	0.268
GenotypexSub-region	0.746	0.162	0.756	0.346
Root mean square error	2.85	11.52	1.5	0.94
Coefficient of variation	0.09	0.27	0.16	0.18

**Table 5:** Test of independence between sensory characteristics and study conditions (sex, locality and genotype).

Sensory Trait	Sex		Locality		Genotype	
	Gl	Value	Gl	Valor	Gl	Value
Color	4	9.33	8	69.77 ***	36	44.38
Taste	4	8.37	8	60.77 ***	36	128.52 ***
Texture	4	9.94	8	50.31 ***	36	113.66 ***
Shape	4	5.21	8	88.94 ***	36	72.98 **

** *p* < 0.001, *** *p* < 0.0001 using Pearson’s χ^2^ test.

**Table 6:** Relative frequency of consumer perception (based on a 1–5 scale) in a hedonic test conducted in three different locations. Colour scale used to indicate with red and related red-colour high values and dark green and green-related colours to show low values.

Trait	Location	Hedonic Score				
		1	2	3	4	5
Color	Cereté	0.19	0.07	0.20	0.22	0.32
	Codazzi	0.18	0.19	0.30	0.20	0.14
	Sevilla	0.09	0.07	0.17	0.30	0.37
Flavor	Cereté	0.22	0.17	0.11	0.22	0.28
	Codazzi	0.17	0.32	0.14	0.24	0.13
	Sevilla	0.20	0.24	0.25	0.22	0.09
Textture	Cereté	0.16	0.18	0.01	0.27	0.28
	Codazzi	0.17	0.35	0.14	0.22	0.12
	Sevilla	0.21	0.24	0.19	0.22	0.14
Shape	Cereté	0.05	0.06	0.18	0.25	0.46
	Codazzi	0.06	0.14	0.33	0.25	0.22

**Table 7:** Contrasts (logistic regression of probability) comparing GM3426-5 with commercial checks (top two rows) and other experimental genotypes.

Genotype	Contrasted with	Flavor		Texture		External Shape	
		Estimator	*p*-Value	Estimator	*p*-Value	Estimator	*p*-Value
GM3426-5	ICA-Costeña	10.206	0.1335	11.181	0.0879	0.63299	0.8250
	Venezolana	0.2432	10.000	−0.3035	0.9996	−139.704	0.0723
	GM3518-42	−11.217	0.0021	−12.810	0.0002	−0.34355	0.9633
	GM3518-66	−0.7914	0.1118	−11.061	0.0026	−0.27431	0.9927
	GM5146-19	−21.115	<0.0001	−20.884	<0.0001	−0.72039	0.3072
	GM5177-3	−21.688	<0.0001	−21.518	<0.0001	−0.35477	0.9606
	GM5194-13	−0.8262	0.1308	−0.7732	0.2170	0.31922	0.9858
	SM3536-44	−0.5553	0.6018	−0.5118	0.6911	−0.77110	0.1492
	SM3767-84	−16.508	<0.0001	−14.855	<0.0001	−0.35148	0.9744

## Data Availability

All the relevant data are available in the manuscript itself. Additional data can be provided on request.
